# Social justice now for an equitable tomorrow: Reflections from the Consortium of Universities for Global Health Conference 2022

**DOI:** 10.1371/journal.pmed.1003995

**Published:** 2022-04-22

**Authors:** Beryne Odeny, Callam Davidson

**Affiliations:** Public Library of Science, San Francisco, California, United States of America

## Abstract

PLOS Medicine editors Beryne Odeny and Callam Davidson report from the Consortium of Universities for Global Health conference.

“Healthy People, Healthy Planet & Social Justice,” was the theme of the second virtual Consortium of Universities for Global Health (CUGH) 2022 conference, held from March 28 to April 1, 2022. In the face of escalating global health and security challenges, this bold theme and the associated agenda (https://cugh.confex.com/cugh/2022/meetingapp.cgi) were welcomed with great anticipation by thousands of stakeholders from 135 countries across the globe. As adeptly put by Dr. Peter A Singer, Special Advisor to the Director General of WHO, the fundamental question at the heart of social justice is simple: “Do we value every human life equally?”. In answering this question, we must acknowledge that what we now consider to be the discipline of global health is in fact anchored by deep and tortuous colonialist roots that continue to bear the fruits of injustice to this day. Over the course of the conference, speakers conceptualized a human rights framework for rethinking global health. This perspective piece presents a curated synopsis of the main CUGH conference and preceding satellite sessions.

The conference commenced with a call from Thuli N. Madonsela, Former Public Protector of South Africa, to uphold the sacrosanct respect for human life given the interconnectedness of humanity’s existence on our shared planet. Her insights on social justice as interpreted within the framework of Ubuntu philosophy brought a breath of fresh air to the debate on neo-colonialism. Thuli’s keynote concluded with an optimistic outlook: “Investing in justice today is like throwing a javelin into the future, one that will become the guardrail for sustainable development.”

Globalization has brought tremendous advances in industry, commerce and trade, and eye-watering financial gains for some in both high income countries (HICs) and low- and middle-income countries (LMICs). Alongside these gains, global openness has contributed to the swift spread of the most formidable maladies of the present day, not least of which are the dual epidemics of COVID-19 and non-communicable diseases (NCDs), climate change and global warming, global corruption, conflict and wars, and ensuing humanitarian crises [1,2]. Pervasive health inequities which compound the toll of these calamities are a stark reminder of how global health has failed the most vulnerable. The COVID-19 pandemic continues to be the litmus test against which our truest values are tested. “If we can’t handle COVID-19, what does it mean about our approach to tackling climate change?”–this was a germane question from a speaker reflecting on inequitable vaccine distribution [3,4].

Comparable to other health sectors awash with global funding, the global COVID-19 vaccine delivery effort has created fertile ground for corruption, due to a toxic combination of high commodity demand, unprecedented resource allocation, and perennially weak health systems with fragmented supply chains [5,6]. It has been shown that corruption can fuel vaccine hesitancy by creating suspicion and mistrust in science and government. This has been witnessed in some countries in Asia, despite commendable levels of vaccine coverage. In contrast, the long-standing National Immunization Program in Brazil created a culture of vaccination and helped minimize hesitancy (despite the influence of the country’s present leadership) [7]. Other factors beyond vaccine-specific factors include contextual, individual, and group influences that can inform hesitancy; these additional factors can be exploited to undergird vaccine efforts–barbers delivering vaccines, and outreach efforts by Buddhist monks, for example.

Beyond the COVID-19 pandemic is the rise of Commercial Determinants of Health (CDoH). CDoH refer to approaches used by corporate sectors to promote products that are detrimental to health [8]. These products include processed foods and drinks, alcohol, and tobacco–factors that are fueling the rising burden of NCDs–more so in LMICs and among the socially disadvantaged in HICs, who bear the largest brunt of related mortality [9]. Unrestrained access to and use of harmful products such as heavy metals and asbestos, pose a threat to poor and vulnerable communities in proximity to mines and industries. These injustices are propagated by powerful corporates that stealthily evade restrictive public health policies to protect their profit margins [10]. A downstream impact of the surge of NCDs in LMICs, is the intense suffering among those dying from terminal illnesses due to the unethical lack of access to palliative care. There is a dearth of palliative resources, including trained health providers, particularly in low resource contexts such as fragile and conflict settings, and among ethnically diverse groups in HICs [11].

The COVID-19 and NCDs conundrums are accompanied by another global health woe–namely the paternalist nature of HIC support for LMICs. Paternalistic support presents in the form of tied aid and technical support which have been used to determine the seat of power, with regard to who holds the money, who generates knowledge, who practices, who publishes, and, ultimately, who thrives in the global health ecosystem. This is demonstrated by institutionalized power asymmetries across funding, academic research, and global health priority setting, which disproportionately favor researchers from HICs at the expense of those from LMICs. To date, less priority has been accorded to health issues of concern, beyond infectious diseases, in the poorest parts of the world such as cancer among other NCDs. Conditionality and increased vertical funding have been shown to limit LMICs’ autonomy to finance their primary health challenges and are linked to reduced government health expenditure with commensurate increases in out-of-pocket/ household expenditure. The health sector is known to be highly corrupt as well as it is well-resourced (accounting of 10% of overall GDP spending) [12]. The lethal mixture of politics, power, and corruption in LMICs is a brewing pot for injustice as it perpetuates a vicious cycle of poverty and disease among the most vulnerable.

Tackling corruption at international and national levels requires multisectoral attention to wider issues of global security, giving people a voice and providing the backing of legal frameworks, to demand accountability and transparency without fear of retaliation. Empowering global health stakeholders and civil societies to engage corporate and political sectors in planetary and global health discourse is an essential tool for fostering health equity, environmental justice, and social justice in business paradigms [2,8]. In this way, leaders can be enlightened and held accountable for performance of equity-based indicators e.g., proportion of specific global goods going to LMICs. Within the global health fraternity, decolonizing global health through inclusive partnerships is necessary to remove longstanding hierarchies in decisional spaces, and shift the balance of power so that more indigenous community actors can define their problems and find relevant solutions [13]. Inspirational stories of the national COVID-19 taskforce in Uganda demonstrate how active communities can promote vaccine uptake [14]. Scaling up community-led integrated health care efforts can extend beyond the pandemic and may even accelerate realization of the UN Sustainable Development Goals. Sustainable funding streams, training, and capacity development to create a robust workforce and enabling environments to host research in LMICs should be at the center of the global health agenda. Other considerations would include leveraging integrated digital and information systems that foster inclusion of marginalized populations in program planning and service delivery, and in so doing uphold equity and inclusion in health system strengthening globally.

Decolonizing global health and upholding social justice will be crucial to containing the impending NCD tsunami, pandemics beyond COVID-19, and climate change. However, throwing off the pernicious colonial legacy presents one of the biggest challenges in global health. No one is exempt from the experience of neo-colonialism regardless of location; thus, all hands are needed on deck to disrupt and resist its existence. Dr. Madhukar Pai of McGill University in Montreal, Canada, and colleagues emphasized that allyship is invaluable to this end–it seeks to identify what the most privileged can do to elevate the voices of those suffocating under the weight of injustice [15]. Beyond speaking up against inequities, meaningful allyship needs disruptive change, sometimes as far as ceding positions of power. The global health community is at a crossroads, a defining moment since its existence, and needs to decide which way to proceed–whether to remain passive to entrenched notions of polarization or to embrace a disruptive paradigm shift that defends social justice and secures sustainable development for all. The question remains–are we ready to shift?

## References

[1] Novotny TE, Mordini E, Chadwick R, Pedersen JM, Fabbri F, Lie R, et al. Bioethical Implications of Globalization: an international consortium project of the European Commission. PLoS Med. 2006;3: e43. doi: 10.1371/journal.pmed.0030043 16420098 PMC1351155

[2] Hotez PJ, Peiperl L. Noncommunicable Diseases: A Globalization of Disparity? PLoS Med. 2015;12: e1001859. doi: 10.1371/journal.pmed.1001859 26218734 PMC4517928

[3] Editors TPM. Vaccine equity: A fundamental imperative in the fight against COVID-19. PLOS Med. 2022;19: 1–4. doi: 10.1371/journal.pmed.1003948 35192620 PMC8863246

[4] Hassan F, London L, Gonsalves G. Unequal global vaccine coverage is at the heart of the current covid-19 crisis. BMJ. 2021;375: n3074. doi: 10.1136/bmj.n3074 34903557

[5] United Nations. Corruption and COVID-19. [Accessed April 14, 2022]. Available: https://www.unodc.org/unodc/en/corruption/covid19.html

[6] Teremetskyi V, Duliba Y, Kroitor V, Korchak N, Makarenko O. Corruption and strengthening anti-corruption efforts in healthcare during the pandemic of Covid-19. Med Leg J. 2021;89: 25–28. doi: 10.1177/0025817220971925 33331228

[7] Domingues CMAS, Maranhão AGK, Teixeira AM, Fantinato FFS, Domingues RAS. The Brazilian National Immunization Program: 46 years of achievements and challenges. Cad Saude Publica. 2020;36 Suppl 2: e00222919. doi: 10.1590/0102-311X00222919 33111749

[8] Kickbusch I, Allen L, Franz C. The commercial determinants of health. Lancet Glob Health. 2016;4: e895–e896. doi: 10.1016/S2214-109X(16)30217-0 27855860

[9] World Health Organization. Noncommunicable diseases.Factsheet. Geneva, Switzerland. Updated in 2021; [Accessed April 14, 2022]. Available: https://www.who.int/news-room/fact-sheets/detail/noncommunicable-diseases

[10] Allen LN, Wigley S, Holmer H. Assessing the association between Corporate Financial Influence and implementation of policies to tackle commercial determinants of non-communicable diseases: A cross-sectional analysis of 172 countries. Soc Sci Med 1982. 2022;297: 114825. doi: 10.1016/j.socscimed.2022.114825 35228150

[11] Knaul FM, Rosa WE, Arreola-Ornelas H, Nargund RS. Closing the global pain divide: balancing access and excess. Lancet Public Health. 2022;7: e295–e296. doi: 10.1016/S2468-2667(22)00063-9 35366402 PMC9245675

[12] García PJ. Corruption in global health: the open secret. Lancet Lond Engl. 2019;394: 2119–2124. doi: 10.1016/S0140-6736(19)32527-9 31785827

[13] Abimbola S, Asthana S, Montenegro C, Guinto RR, Jumbam DT, Louskieter L, et al. Addressing power asymmetries in global health: Imperatives in the wake of the COVID-19 pandemic. PLOS Med. 2021;18: e1003604. doi: 10.1371/journal.pmed.1003604 33886540 PMC8101997

[14] The Government of Uganda, 2020. National Community Engagement Strategy for COVID-19 Response. [Accessed April 14,2022]. Available: https://www.redcrossug.org/images/forms/NATIONAL-COVID-19-COMMUNITY-ENGAGEMENT-STRATEGY-300920-V3.pdf

[15] Pai M. Disrupting Global Health: From Allyship to Collective Liberation. [Accessed in April 14, 2022]. Available: https://www.forbes.com/sites/madhukarpai/2022/03/15/disrupting-global-health-from-allyship-to-collective-liberation/?sh=29d8fab24e62

